# Community health workers and accountability: reflections from an international “think-in”

**DOI:** 10.1186/s12939-018-0781-5

**Published:** 2018-05-25

**Authors:** Marta Schaaf, Jonathan Fox, Stephanie M. Topp, Caitlin Warthin, Lynn P. Freedman, Rachel Sullivan Robinson, Sundararaman Thiagarajan, Kerry Scott, Thoko Maboe, Margareth Zanchetta, Ana Lorena Ruano, Maryse Kok, Svea Closser

**Affiliations:** 1 Averting Maternal Death and Disability Program, Heilbrunn Department of Population and Family Health, Mailman School of Public Health, Columbia University, 60 Haven Ave, B3, New York, NY 10032 USA; 2 Accountability Research Center, School of International Service, American University, 4400 Massachusetts Ave NW, Washington, DC 20016 USA; 3 College of Public Health, Medical and Veterinary Sciences, James Cook University, James Cook Dr, Townsville, QLD 4812 Australia; 4 School of International Service, American University, 4400 Massachusetts Ave NW, Washington, DC 20016 USA; 5 School of Health Systems Studies, Tata Institute of Social Sciences, VN Purav Marg, Deonar, Mumbai 400088 India; 6 Independent Consultant, Bangalore, India and Research Associate, Department of International Health, Johns Hopkins Bloomberg School of Public Health, Baltimore, MD USA; 7 Qondisa Institute Training for Community Health Care Workers, Monument Road, Duncanville, Vereeniging, 1939 South Africa; 8 Daphne Cockwell School of Nursing, Faculty of Community Services, Ryerson University, 350 Victoria St, Office POD 474A, Toronto, ON M5B 2K3 Canada; 9 Center for the Study of Equity and Governance in Health Systems, Guatemala and Center for International Health University of Bergen, Bergen, Norway; 10 KIT | Royal Tropical Institute, P.O. Box 95001, 1090 HA Amsterdam, The Netherlands; 11 Middlebury College, 301 Munroe Hall, Middlebury, VT 05753 USA

**Keywords:** Global health, Community health workers, Accountability, Equity, Power relations, Universal health coverage

## Abstract

Community health workers (CHWs) are frequently put forward as a remedy for lack of health system capacity, including challenges associated with health service coverage and with low community engagement in the health system, and expected to enhance or embody health system accountability. During a ‘think in’, held in June of 2017, a diverse group of practitioners and researchers discussed the topic of CHWs and their possible roles in a larger “accountability ecosystem.” This jointly authored commentary resulted from our deliberations. While CHWs are often conceptualized as cogs in a mechanistic health delivery system, at the end of the day, CHWs are people embedded in families, communities, and the health system. CHWs’ social position and professional role influence how they are treated and trusted by the health sector and by community members, as well as when, where, and how they can exercise agency and promote accountability. To that end, we put forward several propositions for further conceptual development and research related to the question of CHWs and accountability.

## Background

In 1981, community health advocate David Werner posed a provocative question: are community health workers (CHWs) becoming lackeys for over-burdened health systems, or, are they positioned to be liberators [1]? Thirty-five years later the question remains relevant, as CHWs are yet again expected to be a fulcrum in ambitious global and national efforts to accomplish universal health coverage, ‘reach the un-reached’ with health services, develop and support community participation and health education, and help patients to manage long-term health conditions [2].

The relationship between CHWs and accountability is one angle to take in answering Werner’s question. CHWs are frequently explicitly mandated or implicitly expected to enhance or embody health sector accountability.

We use the term “accountability” to describe a system of answerability and sanctions [3, 4]. Answerability means that duty bearers provide explanations and justification regarding actions or decisions taken [3, 4]. When these explanations reveal lapses, ‘hard’ sanctions, such as legal or policy penalties, or ‘soft’ sanctions, such as professional opprobrium or negative publicity, are levied [4].

Distinct dimensions of accountability apply to discussion about CHWs. Since accountability involves power relations, it is indispensable to ask in what direction that power is exercised. Who is accountable to whom, both in theory and in reality? In the emerging field of accountability studies, spatial metaphors abound [5]. Accountability can flow downward, from the health sector as duty bearer to the community as rights holders. It can also flow upward, from health care workers to their managers, policymakers, and in some cases, to funders. ‘Mutual accountability’ refers to the notion of accountability among equals, such as CHWs working on the same team. These accountability relationships can relate to the effective delivery of health services, the fulfillment of CHW professional duties, and the respectful treatment of patients and health care workers.

During a ‘think in’, held in June of 2017, a diverse group of practitioners and researchers discussed the topic of CHWs and their possible roles in a larger “accountability ecosystem” [6, 7]. This group was invited by the ‘think in’ organizers – a university program on maternal health, and a university program on accountability research. Most participants identified as researchers in the area of CHW programs or accountability in one of four focus countries: Brazil, India, South Africa, and the United States. With the exception of the United States, these countries have large scale, government-run CHW programs. We intentionally brought together people from the global health community and people from the accountability and governance community, so that we could pool our disciplinary strengths to debate and to articulate research and policy priorities and propositions that would resonate in both fields.

While CHWs are often conceptualized as cogs in a mechanistic health delivery system, at the end of the day, they are people embedded in families, communities, and the health system. CHWs’ social position and professional role influence how they are treated and trusted by the health sector and by community members, as well as when, where, and how they can exercise agency.

Our conceptual starting point for the meeting was that health accountability is attained when governments respect, protect, and fulfill the right to health, and when health sector employees are treated respectfully. From here, we make the following propositions for consideration by the broader health community. These propositions constitute a nascent discussion and research agenda, and were developed based on our discussions at the ‘think in.’ All of the authors on this commentary attended, although not all attendees are authors.

### CHWs are best understood through a truly global health paradigm

In contrast to a traditional understanding of ‘global health’ that is focused on low and middle income countries [8], CHWs address concerns shared by all countries. Acute disparities, fragmented systems of care, community mistrust, and health sector inability to meet the needs of diverse populations are challenges in many countries, irrespective of levels of income. In fact, given the relative breadth, depth, and length of experience with CHWs in low and middle income countries, south to north learning may be productive [9, 10]. “Thinking and working politically” [11] would entail documenting how CHWs make the health system function better in challenging conditions, and using CHW experience as a lens to reaching a broader understanding of how to disrupt intransigent hierarchies in health systems in countries rich and poor.

### It is important to understand CHWs’ role as bridges between the health sector and communities in the larger context of state society relations and public accountability

From the perspective of community members, the health sector often fails to provide responsive care, or any care at all. However, as community members cannot always easily diagnose the drivers of these failures, the causes of the denial of health rights can appear to be a black box.

Most health system problems have multiple causes that are shaped at multiple levels of the health system. Thus, finding solutions and demanding accountability requires pinpointing responsibility and identifying entry points. CHWs may be able to provide insight and offer solutions in some cases. For example, they can help community members to access well-functioning complaint mechanisms and resolve problems that can be mostly attributed to one individual, such as when a health provider treats a patient with disrespect. Providers and midlevel managers may tolerate some degree of advocacy from CHWs, as they recognize CHWs’ helpful role in facilitating community utilization of health services. However, CHWs may be ill-equipped to diagnose or address problems that are shaped by multiple levels of the health system (so-called “vertically integrated problems”), such as supply chain challenges or corruption in assigning health worker postings. Moreover, it may be difficult for CHWs to advocate for strengthened government accountability for the right to health, as they are at or near the bottom of the frontline health worker hierarchy, lack authority, and depend on government employment for survival. While our discussions focused on public sector CHWs, it is important to acknowledge that Non-Governmental Organization (NGO) supported CHWs would face a different set of limitations, and, that their presence could also complicate the ‘ecology’ of CHWs and government accountability.

### CHWs may engage in collective action with other CHWs for their right to decent work; sometimes this can be a win-win strategy that also empowers communities

As one think-in participant put it, “how can CHWs be expected to empower others if they are not empowered themselves?” This raises the question: how can CHWs forge a collective identity and develop capacity for the sorts of collective action necessary to be heard by more powerful actors? Successful collective action would include CHWs’ being heard as advocates on their own behalf as workers, as problem-solvers in the local service delivery process, and as campaigners for more equitable health policy. To advocate for change in their employment conditions and/or in health policy, in some countries CHWs have unionized, formed professional associations, or otherwise engaged the state as a collective actor. In hierarchical government health systems where CHWs occupy low-status positions, collective voice and action may be especially helpful in pushing for change. In India, Accredited Social Health Activists (ASHAs) have staged a number of protests and strikes at both the state and national levels seeking increased wages and government employee status [12–16], and have met some success [17]. In the United States, the Massachusetts Association of Community Health Workers has on two occasions drafted legislation and found a sponsor to introduce it into the House of Representatives; both bills were signed into law [18]. CHWs in Brazil have unionized in several states, and these unions represent CHWs on labor-related issues and advocate for CHW priorities at the state and federal level.

Organized CHWs may constitute a counterweight to state power, foster stronger alliances between CHWs and their communities, and ultimately boost CHW capacity to promote health sector accountability to these communities. However, it is important to note that, here too, tensions may arise. Professionalization does not necessarily foster stronger relations with the community. Indeed, it can take CHWs further away from the community, as they are distinguished from their neighbors by education, salary, status, and political power. This potential challenge raises three analytical questions: 1) What recruitment strategies, training processes, professional norms, and institutional incentives lead CHWs to defend the public interest as well as their own? 2) Could CHW links with broader labor movements and/or struggles for better community health buttress CHW positions in labor and/or public health negotiations? 3) Under what circumstances can governments be persuaded to see CHW organizations and unionization as an opportunity for advancing community health, rather than as a problem best addressed by ensuring the precarity of CHW employment? Generating answers to these questions is crucial to any agenda seeking to deepen and sustain CHW impact, given the potential for state backlash.

### Boundary organizations can bolster CHW ability to reach up into the accountability system to shift power relations

In rare instances, the organizational interface between CHWs and the state is mediated by a boundary organization; these are connected to the government, but may also have the autonomy to question the state. The design, evaluation, and supervision of the CHW program may be led by an organization that itself straddles the state-society interface.

The Mitanin program in Chhattisgarh, India is supported by the most well-known example of a boundary organization. The State Health Resource Center (SHRC) in Chhattisgarh is a quasi-governmental entity which has links to academia. It has a formal role in both CHW program implementation and learning. Because of its official connections to academia, the SHRC is able to produce and utilize sophisticated evidence related to program implementation and impact. Its quasi-governmental status confers some degree of political influence, so that this evidence is more likely to be translated into policy adaptations that help change and strengthen the program over time [19]. Part of this model has been replicated at the national level, where the National Health Systems Resource Center maintains a formally constituted ASHA mentoring group that engages both health activists and academics.

Not all states have such boundary organizations, but governmental agencies outside the health ministry could also play some kind of boundary role. For example, ombudsman offices, national human rights institutions, and other entities have created space for accountability demands [20].

### CHWs may embody and advance an agenda focusing on the proximate biomedical determinants of health, rather than the social and health system determinants of health

CHWs are individuals addressing the health concerns of other individuals in their communities. In some contexts, the vertical organization of services designed to respond to selective health problems results in CHWs addressing just one or two priority health areas, such as family planning, human immunodeficiency virus (HIV), or immunizations. CHWs may also be pushed to become social marketing agents who are compensated based on the volume of health-related products – such as contraceptives - they distribute or sell. In the larger context of state accountability for the right to health of the most marginalized, the individualized CHW health service delivery approach may take pressure off the community and the local government to foster collective efforts that address the social determinants of health. In contexts where CHWs are extension workers addressing individual health concerns and they have little ability to interact with the health sector, focusing on CHWs as potential agents of accountability can reproduce the erroneous assumption that community health is separate from broader processes of political contestation [21]. In brief, if CHWs’ primary role is to provide services that the state is otherwise unable or unwilling to provide, CHWs can hardly contribute to the community capacity for collective action and voice that may be necessary to get the health sector to be more responsive to community needs. In these contexts, it is unrealistic to assume or to state that CHWs fulfill a transformational accountability role.

### CHW agency may be limited, and they may face significant risks if they try to engage in rights claiming

While there may be some space for CHWs to generate accountability, we need to consider the real power dynamics that undercut CHWs’ ability to be agents of accountability. CHWs are often paid meager salaries, and are often women with pressing social and financial obligations to their families. They may prioritize pay, professionalization, and career progression, rather than how they can facilitate downward accountability to marginalized communities [22]. Making CHWs part of an academic conversation on accountability may be at odds with what CHWs themselves think is most important. By considering CHWs as potential agents of collective action for public accountability, are we just adding to their already significant burdens or encouraging undue risks under regimes hostile to autonomous voice?

### State financial and programmatic capacity and approach to health as a public good cannot be ignored in a discussion about CHWs and accountability

The extent to which governments fulfill the right to health depends on many factors, including government capacity at the national and local levels to design, implement, and monitor health programs and service delivery. Accountability efforts may be more successful when they are accompanied by efforts to build state capacity to deliver responsive and equitable health services. Moreover, it is important to acknowledge that governments may understand accountability to be for financial efficiency as much as for the right to health. Focus on financial efficiency has clear implications for the role of CHWs and the accountability they are expected to foment.

## Conclusion

Some of the issues above have been extensively assessed in research and policy; others have not. Regardless, in the current period of austerity, populism, and changing epidemiological patterns, old questions have new resonance. The synthesis of our propositions suggests avenues of research and areas of policy import for donors and program planners. Some of these propositions are relevant well beyond CHWs; for example, questions about how risk affects most efforts to engage in bottom up monitoring and rights claiming. Moreover, cracking a few of these questions might be one step toward a more transformational agenda, paving the way for CHWs to meet the ambitious expectations put before them. Notably, insight into how and when CHWs can engage in collective action that empowers both them and their communities could inform heath policy and planning, labor and other movement building, and funding in countries at all levels of wealth.
